# Role of Yoga in Modulating Vascular Aging in Type 2 Diabetes Mellitus

**DOI:** 10.7759/cureus.72507

**Published:** 2024-10-27

**Authors:** Vijaykumar G Warad, Akhil Reddy Kankanala, Anuja M Kadagud, Jyoti P Khodnapur

**Affiliations:** 1 General Medicine, Shri B M Patil Medical College, Hospital and Research Centre, BLDE (Deemed to be University), Vijayapura, IND; 2 Physiology, Shri B M Patil Medical College, Hospital and Research Centre, BLDE (Deemed to be University), Vijayapura, IND

**Keywords:** arterial stiffness, cardiovascular health, type 2 diabetes mellitus glycemic control, vascular aging, yoga

## Abstract

Background: Diabetes is one of the top 10 global causes of mortality, with considerable contributions to cardiovascular disease (CVD), respiratory illness, and cancer. Yoga is a traditional Indian mind-body practice that has been linked to improved cardiovascular health, diabetic management, and reduced stress.

Objective: The purpose of this study was to assess the effect of yoga on vascular aging in patients with type 2 diabetes mellitus (T2DM).

Material and methods: From September 2021 to July 2024, Shri B M Patil Medical College, Hospital and Research Centre, BLDE (Deemed to be University), in Vijayapura carried out hospital-based prospective research. Two groups of 70 T2DM patients were created: 35 patients in Group Y (yoga practice) and 35 patients in Group NY (routine without yoga). Age, gender distribution, and baseline glycemic control were similar in both groups. HbA1c, postprandial and fasting blood glucose, and vascular aging using a periscope were determined at baseline and three months later as part of the data-gathering process.

Results: Three months later, Group Y's mean random blood sugar (RBS) and HbA1c levels were considerably lower than those of Group NY (p<0.05). Furthermore, there were notable declines in the brachial-ankle pulse wave velocity and the carotid-femoral pulse wave velocity in Group Y, suggesting a decreased arterial stiffness (p<0.05).

Conclusion: The study concludes that yoga practice significantly improves glycemic control and reduces arterial stiffness in patients with T2DM.

## Introduction

Type 2 diabetes mellitus (T2DM) has become one of the major health issues of the 21st century and is one of the most common causes of cancer and respiratory disorders, leading to mortality [1,2]. In 2019 alone, the World Health Organization reports suggest that non-communicable diseases accounted for about three-fourths of all deaths worldwide, with T2DM accounting for 1.6 million fatalities. Furthermore, projections suggest diabetes will account for 592 million deaths by 2035 [3].

Yoga, which is an ancient practice involving both mind and body, has its roots in India, and dates back around 4,000 years [4], has experienced a rise in use in recent decades in both developed and developing nations [5,6], and there is a notable surge in the area of yoga therapy [7]. Yoga, meaning "yoke or union" in Sanskrit, emphasizes the integration of the body, mind, and spirit, promoting balance, harmony, calm, awareness, and, in traditional practices, selflessness and spiritual enlightenment. It is a systematic method for achieving complete mental, physical, and emotional relaxation. Yoga may be able to lessen the detrimental impact that stress-induced sympathetic nervous system activity has on diabetes patients' ability to regulate their metabolism [8].

Diabetes sufferers may now live a life free of complications owing to yoga. This current study measures blood glucose and glycated hemoglobin (HbA1c) to see how yoga practices affect individuals with T2DM. Yoga also helps in reducing both micro- and macrovascular complications by decreasing vascular aging and significantly impacts heart rate, thereby improving cardiovascular disease incidence [9,10].

Limited research has been conducted on the influence of yoga on vascular aging in Indian T2DM patients. Therefore, this study evaluates the impact of yoga on vascular aging in T2DM patients.

## Materials and methods

This prospective hospital-based investigation was conducted at a tertiary care center in Karnataka from September 2021 to July 2024. It involved 70 patients diagnosed with T2DM, excluding those with insulin-dependent diabetes or a history of diabetic complications. The participants, both inpatients and outpatients, were divided into two groups, including 35 patients in each group. One group followed their routine treatment with oral hypoglycemic drugs (Group NY), while the other group combined their medication with a daily 45-minute yoga practice (Group Y). Ethical approval was obtained from the institutional ethics committee on 30/08/2022 bearing IEC number - BLDE(DU)/IEC/751/2022-23.

Data collection

Data collection included measuring fasting blood sugar and postprandial glucose levels, HbA1c, and vascular aging using a periscope, both at the beginning of the study and after three months. Investigations also encompassed complete blood count, fundoscopy, weight, height, and body mass index.

Statistical analysis

G*Power (ver. 3.1.9.4; The G*Power Team, Germany) software was utilized to guarantee a sample size large enough to attain 98% power in identifying the differences between the groups. Categorical and continuous variables were analyzed using chi-square and unpaired t-tests, respectively. P<0.05 was considered statistically significant.

## Results

**Figure 1:**
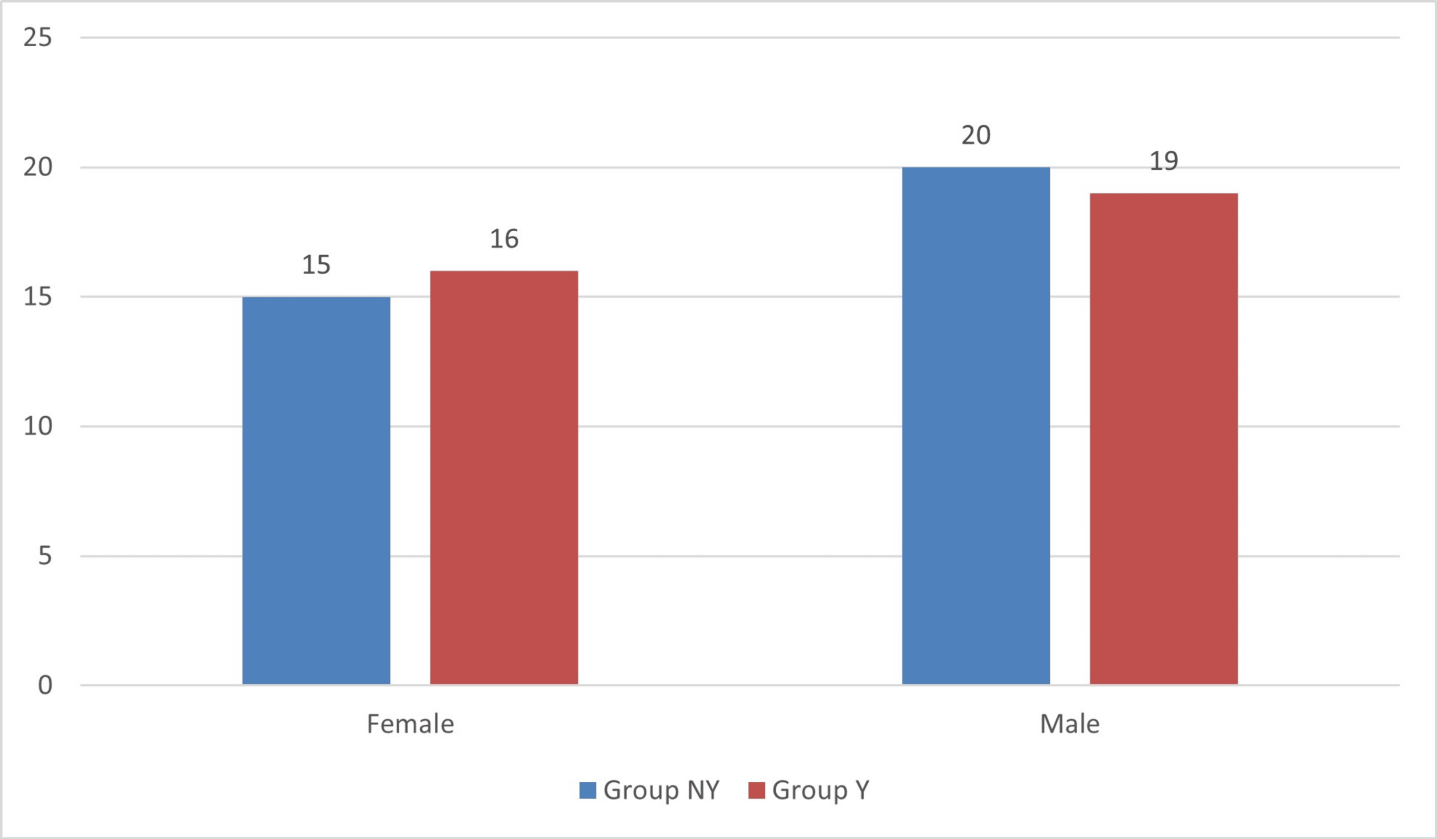
Gender comparison between the groups The bar graph indicates the gender-wise distribution of patients between the groups. The blue bars indicate Group NY, and the red bars indicate Group Y.

In the present study, a total of 70 patients fulfilling the inclusion criteria were included. The patients were divided into two groups: 35 patients in Group Y and 35 patients in Group NY. The demographics are presented in Table 1. Table 2 compares the periscope measurements between the groups at baseline. Figure 1 shows the gender comparison between the groups.

**Table 1:** Comparison of the demographic details and glycemic status between the groups DM - Diabetes Mellitus; SD - Standard Deviation; RBS - Random Blood Sugar; HbA1c - Glycated Hemoglobin * Significant

	Group NY		Group Y		
	Mean	SD	Mean	SD	p-value
Age	51.2	7.4	51.1	7.6	0.975
Duration of DM	5.2	1.7	5.1	1.6	0.884
Height	165.8	6.3	164.9	7.8	0.57
Weight	72.1	8.4	71.3	7.6	0.67
Baseline RBS	235.5	23.7	236.1	25.2	0.926
Baseline HbA1c	7.79	.67	7.80	0.68	0.986
RBS after 3 months	227.2	23.3	206.1	25.2	0.01*
HbA1c after 3 months	7.73	0.67	7.25	0.70	0.01*

**Table 2:** Comparison of the periscope measurement between the groups at baseline SD - Standard Deviation; PR - Pulse Rate; SBP - Systolic Blood Pressure, DBP - Diastolic Blood Pressure; Right ba PWV - Right Brachial-Ankle Pulse Wave Velocity; Left ba PWV - Left Brachial-Ankle Pulse Wave Velocity; CF PWV - Carotid-Femoral Pulse Wave Velocity: R Bra ASI - Right Brachial Arterial Stiffness Index; L Bra ASI - Left Brachial Arterial Stiffness Index; R Ank ASI - Right Ankle Arterial Stiffness Index; L Ank ASI - Left Ankle Arterial Stiffness Index; R ABI - Right Ankle-Brachial Index; L ABI - Left Ankle-Brachial Index; Ao Sys - Aortic Systolic Pressure; Ao PP - Aortic Pulse Pressure; Ao Dia - Aortic Diastolic Pressure; Ao AugP - Aortic Augmentation Pressure; Alx - Aortic Augmentation Index

Baseline	Group NY		Group Y		
	Mean	SD	Mean	SD	p-value
PR	70.0	8.4	70.1	8.2	0.97
SBP	110.8	10.2	108.7	10.3	0.39
DBP	66.3	6.8	64.9	6.3	0.34
Pulse pressure	45.9	5.7	45.0	5.7	0.50
Right ba PWV	1562.0	499.8	1611.6	489.3	0.677
Left ba PWV	1538.3	636.7	1498.4	596.4	0.78
CF PWV	1020.5	463.8	991.9	431.9	0.79
R Bra ASI	22.1	2.6	21.7	3.0	0.53
L Bra ASI	19.0	2.9	19.1	3.5	0.97
R Ank ASI	22.2	14.1	20.7	14.0	0.65
L Ank ASI	34.4	8.5	34.0	8.1	0.85
R ABI	1.07	.15	1.03	.16	0.331
L ABI	1.123	.084	1.115	.077	0.689
Ao Sys	95.6	13.0	93.6	13.5	0.524
Ao PP	30.8	6.9	29.9	7.2	0.601
Ao Dia	64.3	7.3	63.2	6.9	0.502
Ao AugP	7.1	6.5	6.4	6.3	0.655
Alx	21.0	15.3	19.5	14.6	0.661

After three months of intervention, the study documented significantly lower mean levels of the mean random blood glucose and HbA1c among the Group Y patients compared to Group NY patients (p<0.05).

Following the yoga practice intervention, Group Y's mean periscope parameters were determined to be lower than those of Group NY (Table 3). When comparing Group Y to Group NY, there is a notable decrease in the mean level of brachial-ankle and carotid-femoral pulse wave velocity, indicating a considerable stiffness (p<0.05).

**Table 3:** Comparison of the periscope measurement between the groups after three months SD - Standard Deviation; PR - Pulse Rate; SBP - Systolic Blood Pressure, DBP - Diastolic Blood Pressure; Right ba PWV - Right Brachial-Ankle Pulse Wave Velocity; Left ba PWV - Left Brachial-Ankle Pulse Wave Velocity; CF PWV - Carotid-Femoral Pulse Wave Velocity; R Bra ASI - Right Brachial Arterial Stiffness Index; L Bra ASI - Left Brachial Arterial Stiffness Index; R Ank ASI - Right Ankle Arterial Stiffness Index; L Ank ASI - Left Ankle Arterial Stiffness Index; R ABI - Right Ankle-Brachial Index; L ABI - Left Ankle-Brachial Index; Ao Sys - Aortic Systolic Pressure; Ao PP - Aortic Pulse Pressure; Ao Dia - Aortic Diastolic Pressure; Ao AugP - Aortic Augmentation Pressure; Alx - Aortic Augmentation Index *Significant

After 3 months	Group NY		Group Y		
	Mean	SD	Mean	SD	p-value
PR	69.0	8.4	69.1	8.2	0.97
SBP	109.8	10.2	107.7	10.3	0.39
DBP	65.3	6.8	63.9	6.3	0.34
Pulse pressure	44.9	5.7	44.0	5.7	0.50
Right ba PWV	1561.0	499.8	1323.0	471.0	0.01*
Left ba PWV_	1537.3	636.7	1251.3	593.0	0.05*
CF PWV	1019.5	463.8	885.0	425.2	0.01*
R Bra ASI	21.1	2.6	20.7	3.0	0.53
L Bra ASI	16.4	6.1	16.4	6.4	0.98
R Ank ASI	21.2	14.1	19.7	14.0	0.65
L Ank ASI	33.4	8.5	33.0	8.1	0.85
R ABI	.1	.1	.0	.2	0.331
L ABI	.1	.1	.1	.1	0.69
Ao Sys	94.6	13.0	92.6	13.5	0.52
Ao PP	29.8	6.9	28.9	7.2	0.60
Ao Dia	63.3	7.3	62.2	6.9	0.50
Ao AugP	6.1	6.5	5.4	6.3	0.65
Alx	20.0	15.3	18.5	14.6	0.66

## Discussion

In people with T2DM, CVD continues to be the primary cause of illness and mortality [11,12]. This is mostly because of accelerated vascular aging, which is typified by endothelial dysfunction, arterial stiffness, and decreased vasodilation. This accelerated aging process is facilitated by low-grade inflammation, insulin resistance, hyperglycemia, and chronic oxidative stress. Yoga is a traditional Indian mind-body practice that combines physical postures, breathing techniques, and meditation. It is well-known around the world for its positive effects on the heart, reduction of stress, and management of blood sugar levels [8,13]. According to preliminary data, yoga may improve microvascular health through mechanisms such as lowered oxidative stress and inflammation, improved metabolic parameters, and activation of the parasympathetic nervous system, as well as by improving endothelial function and lowering arterial stiffness in T2DM patients [14].

In this study, 70 T2DM patients were placed into two groups: 35 who practiced yoga (Group Y) and 35 who continued their regular routine without yoga (Group NY). The groups were comparable in age and gender distribution, though there was a slight male preponderance. Both groups showed similar baseline glycemic control and vascular aging parameters. After three months, Group Y demonstrated significantly lower mean random blood glucose and HbA1c levels compared to Group NY. Furthermore, Group Y's carotid-femoral and brachial-ankle pulse wave velocities were significantly reduced, suggesting a reduction in arterial stiffness.

These findings align with previous studies by Dash et al. [15], Jain et al. [16], and Beena et al. [17], which documented significant improvements in fasting blood glucose, HbA1c, plasma triglycerides, cholesterol levels, and reduced cortisol and ferritin levels among patients practicing yoga. Yoga practice also showed benefits in reducing anxiety, blood pressure, total cholesterol, and stress, indicating its potential as a lifestyle intervention for managing type 2 diabetes and improving psychological well-being. Yoga's role in lowering blood sugar levels and HbA1c helps prevent microvascular and macrovascular complications and improves lipid profiles, reducing the risk of hypertension and coronary artery disease [15]. Yoga as an adjunct to medical therapy can optimize biochemical markers, lower medication doses, enhance physical and mental alertness, and prevent complications in diabetic patients.

## Conclusions

In this study, 70 patients meeting the inclusion criteria were divided into two groups: 35 patients practicing yoga (Group Y) and 35 patients following their routine without yoga (Group NY). Both groups had comparable mean age, gender distribution, duration of diabetes mellitus, height, and weight with no significant differences. Initially, glycemic control parameters, such as RBS and HbA1c, were also similar between the groups. However, after three months, Group Y showed significantly lower mean levels of RBS and HbA1c compared to Group NY (P<0.05). Baseline measures of arterial stiffness were similar between the groups (P>0.05), but post-intervention, the mean values of brachial-ankle and carotid-femoral pulse wave velocities were much lower in Group Y, indicating reduced arterial stiffness (P<0.05). The study concludes that practicing yoga significantly improves glycemic control and reduces arterial stiffness in patients with diabetes mellitus.
